# Analysis of neurovascular unit after voluntary physical exercise in a transgenic mouse of Alzheimer’s disease

**DOI:** 10.1002/alz.087970

**Published:** 2025-01-03

**Authors:** José de Jesús Andrade, Erika Orta‐Salazar, Sofia Díaz‐Cintra, Luis Oskar Soto‐Rojas

**Affiliations:** ^1^ Universidad Nacional Autónoma de México, Querertaro, QA Mexico; ^2^ Instituto de Neurobiología UNAM, Queretaro, QA Mexico; ^3^ Facultad de Estudios Superiores Iztacala, Universidad Nacional Autónoma de México, Carrera de Médico Cirujano, MEXICO CITY, EM Mexico

## Abstract

**Background:**

Alzheimer’s disease (AD) stands out as the most prevalent neurodegenerative condition globally, marked by a progressive cognitive decline. Its distinctive histopathological features include neurofibrillary tangles composed of Tau protein aggregates and amyloid beta (Aβ) aggregates forming neuritic plaques in the parenchyma and cerebral amyloid angiopathy (CAA) in blood vessels. Intriguingly, it has been hypothesized that CAA induces alterations in the cells comprising the neurovascular unit (NVU), exacerbating the disease’s symptoms. Currently, effective treatments for AD remain elusive. The triple transgenic mouse model for AD (3xTg‐AD), harboring three mutations associated with familial AD (*PS1M146V*, *APPSWE*, and *tauP301L*), is widely employed to investigate molecular mechanisms and assess therapeutic strategies. One promising non‐pharmacological approach is physical exercise, a planned, repetitive, and regulated physical activity. However, its impact on the cerebrovascular system remains unclear. This study aims to analyze the effect of physical exercise on the NVU in the 3xTg‐AD model.

**Method:**

A total of 40 ten‐month‐old female mice, including 3xTg‐AD (n = 20) and non‐Tg (n = 20), were equally divided into exercise and sedentary groups. A voluntary physical exercise intervention spanning three months, with a frequency of five times a week, was implemented. Subsequently, we evaluated cognitive functions, CAA, and NVU morphostructure through histological assays

**Result:**

Our results demonstrate that 3xTg‐AD mice develop vascular amyloid deposits, correlating with NVU alterations and cognitive deficits. Intriguingly, voluntary physical exercise reduces CAA and amyloid plaques, coinciding with improvements in cognitive abilities and NVU component integrity.

**Conclusion:**

The intervention with voluntary physical exercise holds promise for enhancing vascular system stability and reducing CAA in symptomatic stages of AD, presenting a potential therapeutic avenue for individuals affected by the disease.

